# Accuracy and safety of robot-assisted cortical bone trajectory screw placement: a comparison of robot-assisted technique with fluoroscopy-assisted approach

**DOI:** 10.1186/s12891-022-05206-y

**Published:** 2022-04-06

**Authors:** Yue Li, Long Chen, Yuzeng Liu, Hongtao Ding, Hongyi Lu, Aixing Pan, Xinuo Zhang, Yong Hai, Li Guan

**Affiliations:** 1 Department of Orthopedic Surgery, Beijing Chao-Yang Hospital, Capital Medical University, GongTiNanLu 8#, Chaoyang District, Beijing, 100020 China; 2 Department of Orthopedics, Guangzhou Red Cross Hospital Affiliated To Jinan University, No. 396, Mid Tongfu Road, Haizhu District, Guangzhou, 510000 Guangdong P. R. China

**Keywords:** Cortical bone trajectory, Robot-Assisted, Accuracy, Learning curve, Facet joint violation

## Abstract

**Objective:**

To compare the safety and accuracy of cortical bone trajectory screw placement between the robot-assisted and fluoroscopy-assisted approaches.

**Methods:**

This retrospective study was conducted between November 2018 and June 2020, including 81 patients who underwent cortical bone trajectory (CBT) surgery for degenerative lumbar spine disease. CBT was performed by the same team of experienced surgeons. The patients were randomly divided into two groups—the fluoroscopy-assisted group (FA, 44 patients) and the robot-assisted group (RA, 37 patients). Robots for orthopedic surgery were used in the robot-assisted group**,** whereas conventional fluoroscopy-guided screw placement was used in the fluoroscopy-assisted group. The accuracy of screw placement and rate of superior facet joint violation were assessed using postoperative computed tomography (CT). The time of single screw placement, intraoperative blood loss, and radiation exposure to the surgical team were also recorded. The χ^2^ test and Student’s t-test were used to analyze the significance of the variables (*P* < 0.05).

**Results:**

A total of 376 screws were inserted in 81 patients, including 172 screws in the robot-assisted group and 204 pedicle screws in the fluoroscopy-assisted group. Screw placement accuracy was higher in the RA group (160, 93%) than in the FA group (169, 83%) (*P* = 0.003). The RA group had a lower violation of the superior facet joint than the FA group. The number of screws reaching grade 0 in the RA group (58, 78%) was more than that in the FA group (56, 64%) (*P* = 0.041). Screw placement time was longer in the FA group (7.25 ± 0.84 min) than in the RA group (5.58 ± 1.22 min, *P* < 0.001). The FA group had more intraoperative bleeding (273.41 ± 118.20 ml) than the RA group (248.65 ± 97.53 ml, *P* = 0.313). The radiation time of the FA group (0.43 ± 0.07 min) was longer than the RA group (0.37 ± 0.10 min, *P* = 0.001). Furthermore, the overall learning curve tended to decrease.

**Conclusions:**

Robot-assisted screw placement improves screw placement accuracy, shortens screw placement time, effectively improves surgical safety and efficiency, and reduces radiation exposure to the surgical team. In addition, the learning curve of robot-assisted screw placement is smooth and easy to operate.

## Background

In 2009, Santoni et al. introduced a new method for screw insertion called cortical bone trajectory (CBT) [1]. This new trajectory follows a caudal-to-head path in the sagittal plane and a lateral path in the transverse plane. The unique trajectory of the CBT technique increases the contact area of the screw threads with the bone cortex, while allowing smaller incisions and muscle stripping [1, 2]. Compared to conventional techniques, CBT increases screw purchase while reducing surgical trauma. Therefore, the CBT technique is considered appropriate for cases of severe obesity, osteoporosis, and those requiring revision of conventional pedicle screws [3].

CBT has been shown to be a safe and valuable option for screw fixation in spinal surgery. Level 2 and 3 clinical studies have demonstrated equal clinical and radiographic outcomes and lower perioperative complication rates than conventional techniques [4]. As the CBT technique is becoming more accepted, an increasing number of surgeons are performing posterior lumbar fusion with the CBT technique in eligible patients. However, it can be challenging for less experienced surgeons to determine the optimal entry point and the unique trajectory of CBT. Even for experienced surgeons, smaller incisions and degenerated facet joints affect the accuracy of screw placement [5]. Since the insertion point of the CBT is closer to the spinal canal and nerve than that of the conventional trajectory, the adjacent neural tissue can easily be damaged after inserting the screw at an incorrect angle [6]. Screw misplacement can violate the superior facet joint, causing adjacent degeneration, postoperative decline in quality of life, and low back pain [7, 8].

Navigation or robot-assisted screw placement can be used to improve screw placement accuracy. The accuracy of robot-assisted screw placement has been demonstrated in previous studies [9, 10]. Particularly, the scale proposed by Gertzbein et al. [11] is mostly used by studies evaluating the accuracy of CBT screw placement. This scale is mainly applicable to pedicle screw techniques, but it is often unable to accurately assess the unique screw path of CBT. Given this, a specific CBT score proposed by Ding et al. [12] was used to assess the trajectory of robot-assisted CBT in the present study. Herein, we performed CBT using TiRobot (TINAVI Medical Technologies Co., Ltd., Beijing, China) robot-assisted and fluoroscopy-assisted (FA) screw placement in 37 and 44 patients, respectively. We then evaluated the accuracy and safety of robot-assisted CBT screw placement compared to fluoroscopy-assisted screw placement in three aspects: screw placement accuracy, superior facet joint violation, and intraoperative radiation exposure.

## Methods

### Eligible criteria

This study reviewed patients who underwent CBT for degenerative lumbar spine disease between November 2018 and June 2020. The inclusion criteria were as follows: (I) age ≤ 85 years, (II) diagnosis of degenerative lumbar disease that required surgery, and (III) need for the cortical trajectory screw technique for screw placement. Meanwhile, the exclusion criteria were as follows: (I) patients with scoliosis > 30°; (II) patients with infection, trauma, or severe psychosis; and (III) patients with vertebral body dysmorphism affecting screw placement, such as severe lumbar degeneration.

### Fluoroscopy-assisted implantation

Patients were placed in the prone position, and the surgical site was determined using fluoroscopy. An approximately 5-cm midline skin incision was made. The muscles were then separated layer by layer to expose the surgical site, and screw trajectories followed the mediolateral and caudal-directed paths. The entry point was located at the junction of the midpoint of the superior facet joint process 1 mm below the lower edge of the transverse process. After confirming correct placement with anteroposterior and lateral fluoroscopy, the guidewire for positioning was used to perform screw placement by hand. Decompression was performed as appropriate after the completion of screw placement.

### Robot-assisted implantation

The TiRobot consists of a surgical plan, an optical tracking unit, a control workstation, and a surgical robotic arm that has six degrees of freedom (6-DOF) to allow accurate and stable positioning.

**Fig. 1:**
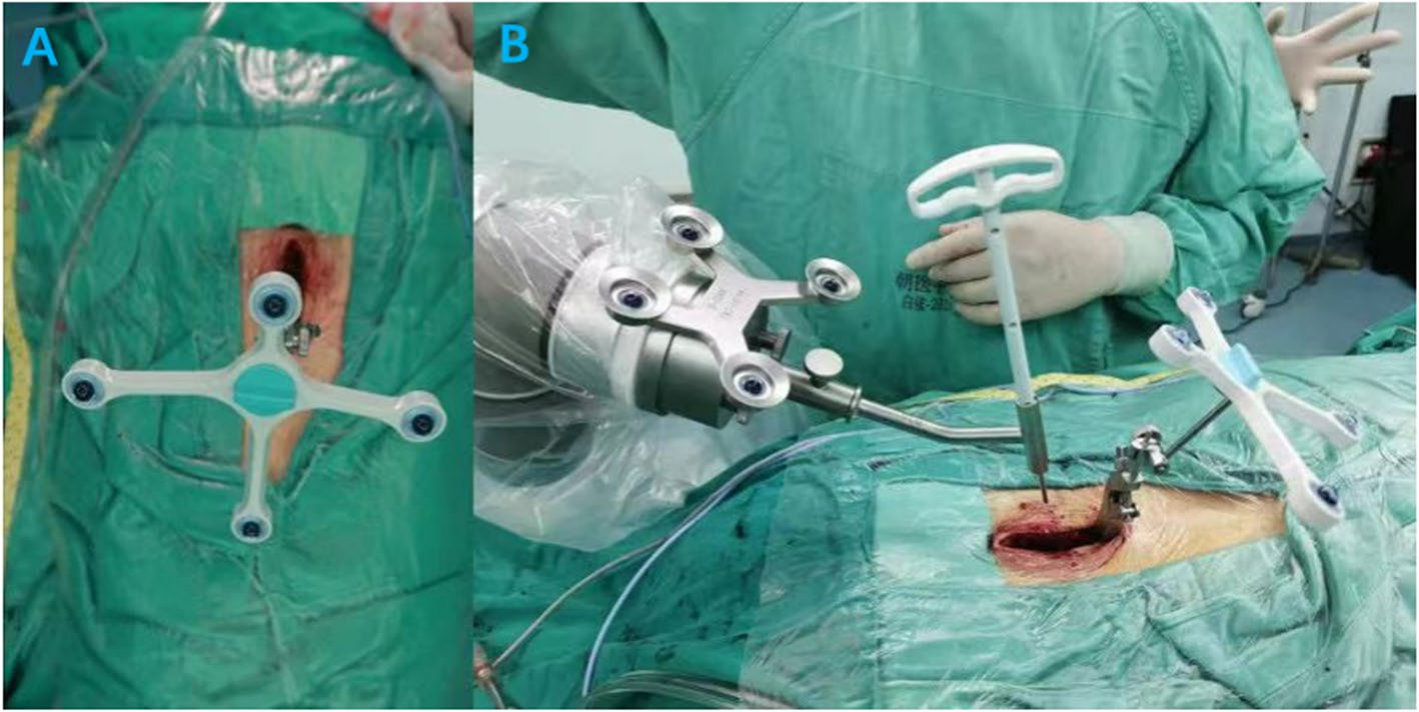
**A** Positioning with infrared. **B** The screw is placed under the assistance of robotic arm, according to the pre-planned path

Patients were placed prone on a robot-assisted specialized operating table, with the tracker positioned over the spinous process at the level of the operative vertebral body. The calibration device was fixed by the robotic arm on the skin as close to the surgical site as possible. Three-dimensional images of the screw placement area were then acquired using a C-arm scanner (Siemens Medical Solutions; Erlangen, Germany) (Fig. 1). The registration was performed by the automatic identification of the calibration device, and the screw trajectories were planned using the TiRobot system. Afterwards, screw placement was performed along the planned path at the planned entry point. Real-time navigational monitoring was used to monitor drilling, and screw placement was subsequently completed. Intraoperative fluoroscopy was required following robot-assisted screw placement to continuously correct the trajectory of the screw until ideal.

### Parameter measurement

The time for single screw placement, intraoperative blood loss, and radiation exposure to the medical team were recorded. The time for single screw placement was defined as the total time for satisfactory screw placement for all screws divided by the total number of screws. Robot-assisted screw placement included screw path planning and screw placement time, whereas fluoroscopy-assisted screw placement included guidewire positioning and screw placement time. The time to adjust for an unsatisfactory trajectory indicated by intraoperative fluoroscopy was also accounted for. Radiation exposure to the medical team was measured using total intraoperative fluoroscopy time.

Screw placement accuracy was evaluated using the score proposed by Ding et al. [12] Their method includes the following grades. Grade 0 was classified when the screw passes completely within the pedicle cortical canal or the cortical screw, with a tiny breach of the medial or lateral pedicle cortex (< 1/2 of the screw diameter passes the medial pedicle cortex) on axial and sagittal computed tomography (CT). Moreover, the tip penetrating the vertebrae should not penetrate the pedicle cortex into the adjacent neural foramen nor should it penetrate the upper endplate into the intervertebral disc. Grade 1 included medial cortical bone perforation (MCP) and lateral cortical bone perforation (LCP). On axial CT, the screw should partially perforate the medial or lateral pedicle cortex (> 1/2 of the screw diameter passes through the medial pedicle cortex). Lastly, Grade 2 included MCP, LCP, anterior cortical bone perforation (ACP), endplate perforation (EPP), and foraminal perforation (FP). On axial CT, the screw should completely perforate the medial or lateral pedicle cortex and the anterior cortex of the vertebral body. In addition, sagittal CT images in Grade 2 should show that the screw perforates the foramen or the screw tip perforates the upper endplates into the disc.

**Fig. 2:**
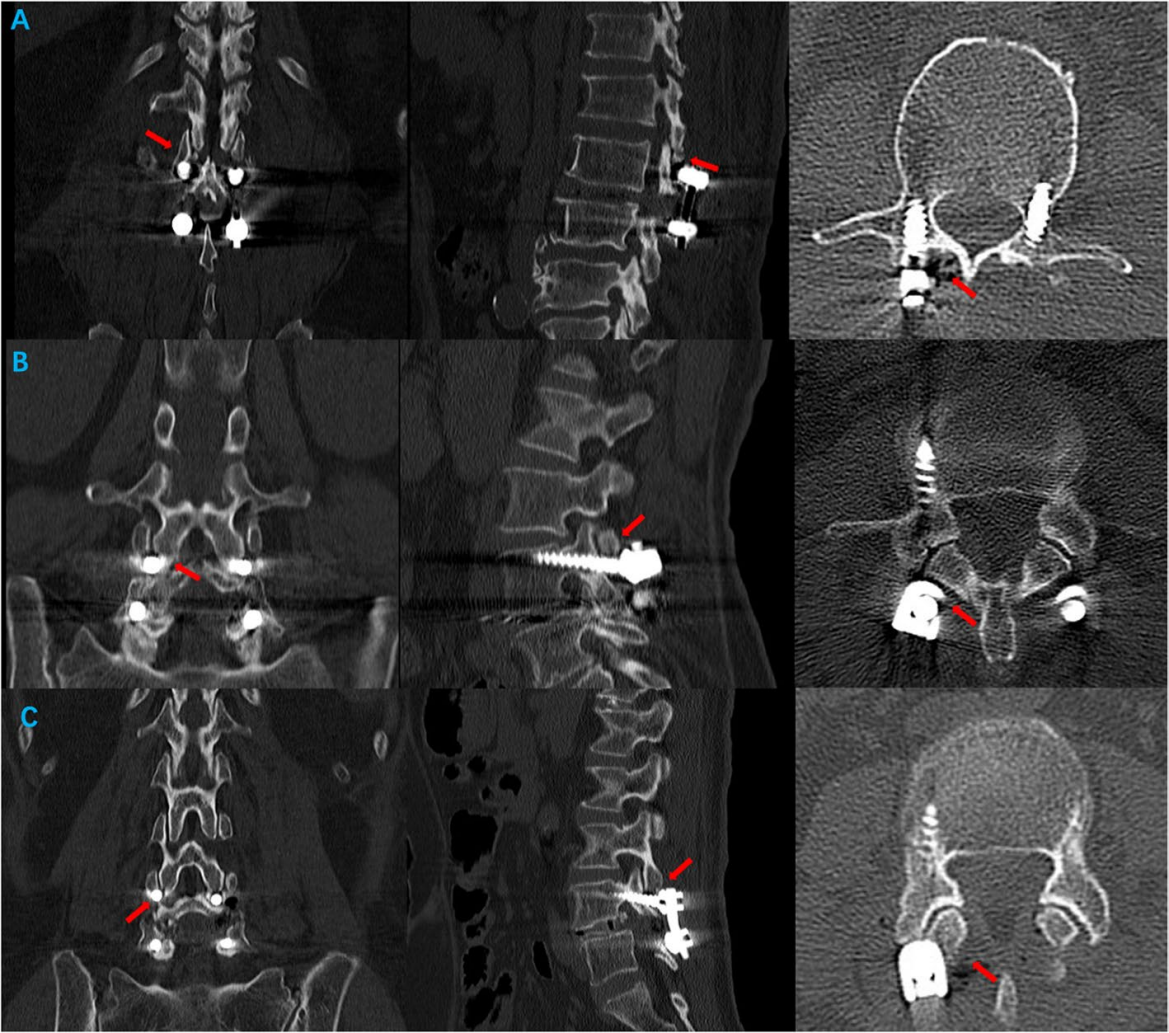
Facet joint violation classification, as described by Yson et al. [13] and Moshirfar et al. [14]

Facet joint violation was evaluated according to the classification described by Yson et al. [13] and Moshirfar et al. [14] on axial, sagittal, and coronal CT. Grade 0 was classified when the superior facet joints were not violated (Fig. 2A). Grade 1 was classified when the screw head, screw shank, or rod was within 1 mm of the superior facet joint but not into the superior facet joint (Fig. 2B). Lastly, Grade 2 was classified when there was definite violation of the screw head, screw shaft, or rod into the superior facet joint (Fig. 2C).

In this study, screws with a length of 35 mm and a diameter of 5.5 mm were used in both groups. Patients in both groups routinely underwent CT scanning before and after surgery, and the CT in our center was performed using GE CT machine (Milwaukee, Wisconsin, USA), with scanning parameters of interval (mm) 0.625, gantry tilt S0.0, kV 120, and mA 450.

### Statistical analysis

The SPSS version 21.0 was used to analyze the collected data. All values were expressed as means ± standard deviation. The χ^2^ test was used for unquantified data, and Student’s t-test was used to compare differences between groups. Statistical significance was set at *P* < 0.05.

## Results

A total of 81 patients were included in this study, with an average of 4.64 ± 0.94 screws placed per patient. There was no statistically significant difference in the number of screws placed between the two groups. A total of 172 screws were placed in 37 patients who underwent robot-assisted CBT screw placement, whereas 204 screws were placed in 44 patients who underwent fluoroscopy-assisted CBT screw. There were no significant differences in parameters, including sex, age, and body mass index (BMI), between the two groups.

Screw placement time was longer in the FA group (7.25 ± 0.84 min) than in the RA group (5.58 ± 1.22 min, *P* < 0.001). The FA group had more intraoperative bleeding (273.41 ± 118.20 ml) than the RA group (248.65 ± 97.53 ml, *P* = 0.313). The radiation time of FA group (0.43 ± 0.07 min) was longer than in the RA group (0.37 ± 0.10 min, *P* = 0.001). No patients in the RA group underwent revision surgery, while three patients in the FA group underwent revision surgery due to neurological injury caused by screw misplacement. The symptoms of these patients were relieved after surgery. There were also no statistically significant differences in revision and neurological injury complication rates between the two groups. Furthermore, there were four patients who developed infectious complications, with one patient in the FA group and three patients in the RA group, although no statistically significant difference was found (Table 1).

**Table 1:** Basic information and clinical parameters

Variable	Total (*n* = 376)	RG (172)	FH (204)	*P*
Patients (n)	81	37	44	
Sex				0.778
Male		14	18	
Female		23	26	
Mean age (years)	63.65 ± 10.50	64.86 ± 9.70	62.64 ± 11.13	0.344
Mean BMI (kg/m2)	26.48 ± 4.38	25.99 ± 4.20	26.90 ± 4.53	0.355
Screws (n) per case	4.64 ± 0.94	4.65 ± 0.95	4.64 ± 0.94	0.954
Time for single screw Placement	6.49 ± 1.33	5.58 ± 1.22	7.25 ± 0.84	*P* < 0.001^*^
Intraoperative blood Loss (ml)	262.10 ± 109.29	248.65 ± 97.53	273.41 ± 118.20	0.313
Radiation time (min)	0.41 ± 0.10	0.37 ± 0.10	0.43 ± 0.07	0.001^*^
Revision	3	0	3	0.108
Complications				
Infection	4	1	3	0.401
Neurological injury	0	1	3	0.108

*RG* Robot-Assisted, *FH* Free-Hand

^*^P < 0.05. Statistically significant differences between the two groups

A total of 160 screws (93%) and 169 screws (83%) in the RA and FA groups, respectively, achieved Grade 0 (*P* = 0.003). In the RA group, Grade 1 was achieved in 5% of screws, which was lower than that in the FA group (10%) (*p* = 0.058). Moreover, four screws (2%) in the RA group achieved Grade 2, which was fewer than the 15 Grade 2 screws (7%) in the FA group (Table 2). In terms of superior facet joint violation, the number of screws reaching Grade 0 in the RA group was 58 (78%), which was more than in the FA group (56, 64%) (*P* = 0.041). The remaining classifications of the RA group included 12 Grade 1 screws and 4 Grade 2 screws, whereas in the FA group, there were 22 Grade 1 screws and 10 Grade 2 screws (Table 3).

**Table 2:** Accuracy classification for screw trajectory

Grade	RG (*n* = 172)	FH (*n* = 204)	*P*
0	160	169	0.003^*^
1	8	20	0.058
2	4	15	0.027^*^

^*^P < 0.05. Statistically significant differences between the two groups

**Table 3:** Classification of superior facet joint violation

Grade	RG (*n* = 74)	FH (*n* = 88)	*P*
0	58	56	0.041^*^
1	12	22	0.171
2	4	10	0.179

^*^P < 0.05. Statistically significant differences between the two groups

**Fig. 3:**
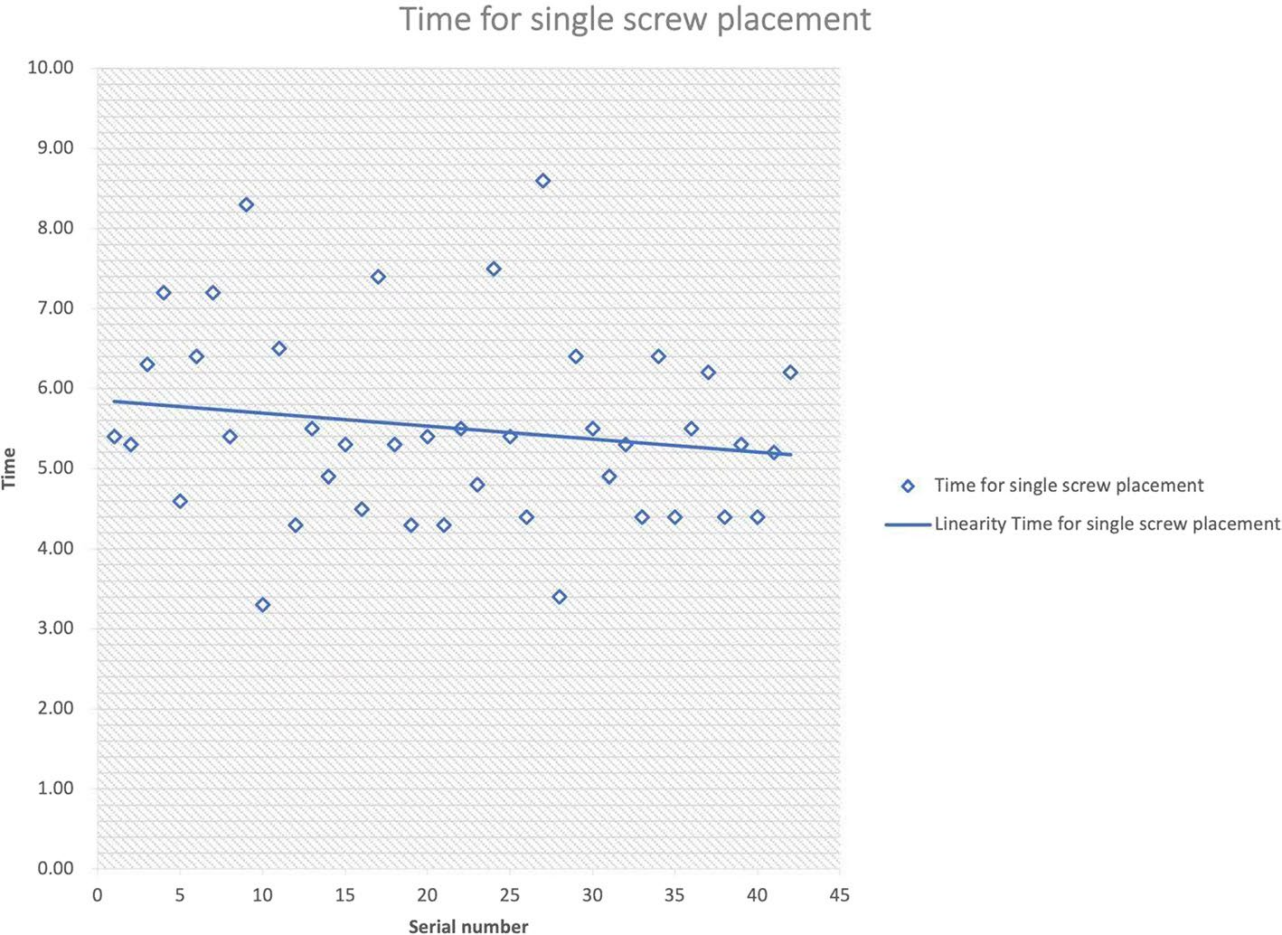
Time for single screw placement

In addition, the time for single screw placement in the first 50% of cases (5.73 ± 1.27 min) was longer than that in the last 50% of cases (5.43 ± 1.20 min, *P* = 0.461). Based on the linear distribution of the scatter diagram, the average screw placement time showed a gradually decreasing trend (Fig. 3).

## Discussion

**Fig. 4:**
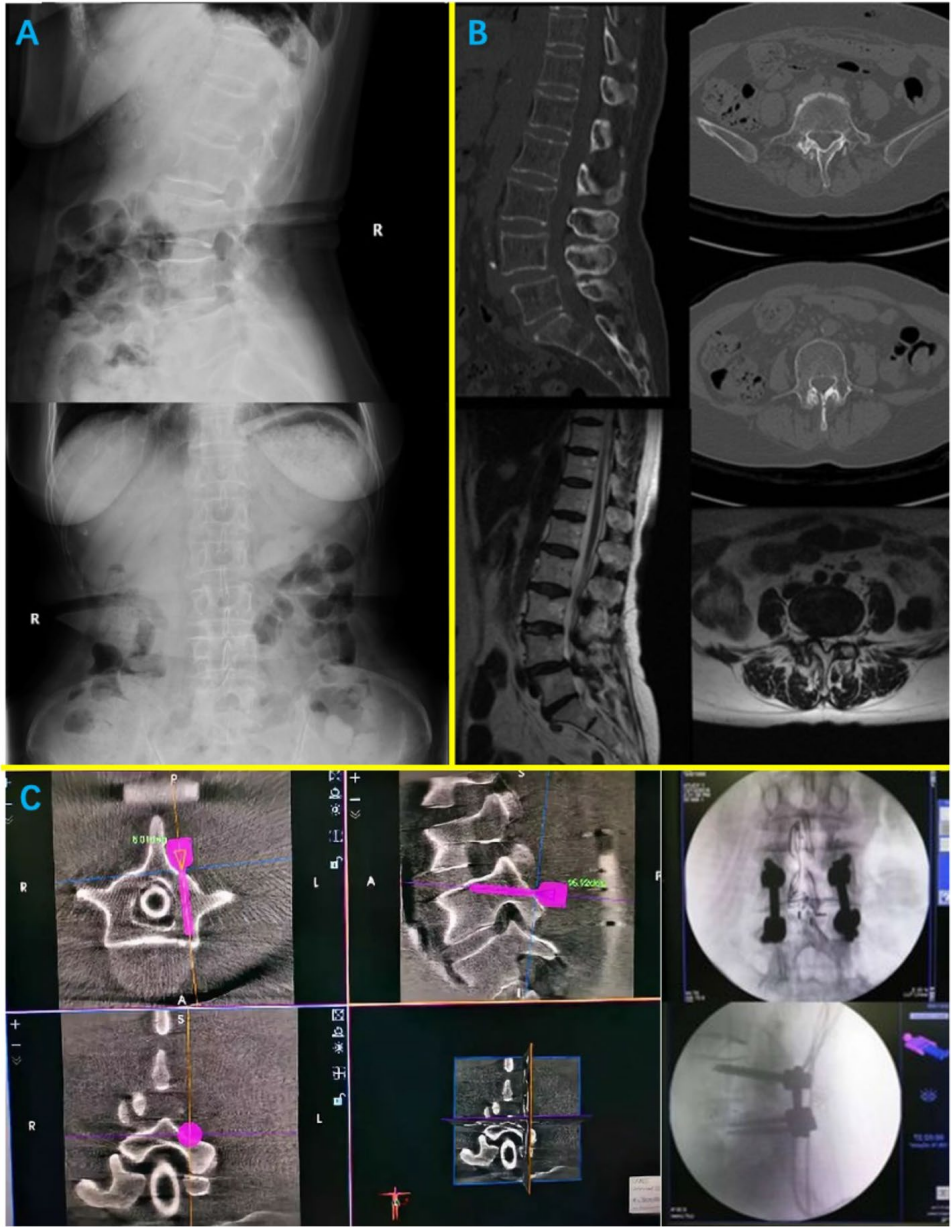
**A** Preoperative X-ray of patient; **B** Preoperative CT showed L4/5 spondylolisthesis facet joints hyperplasia and degeneration, MRI showed L4/5 spinal stenosis. **C** Robot-assisted intraoperative positioning and intraoperative fluoroscopy

**Fig. 5:**
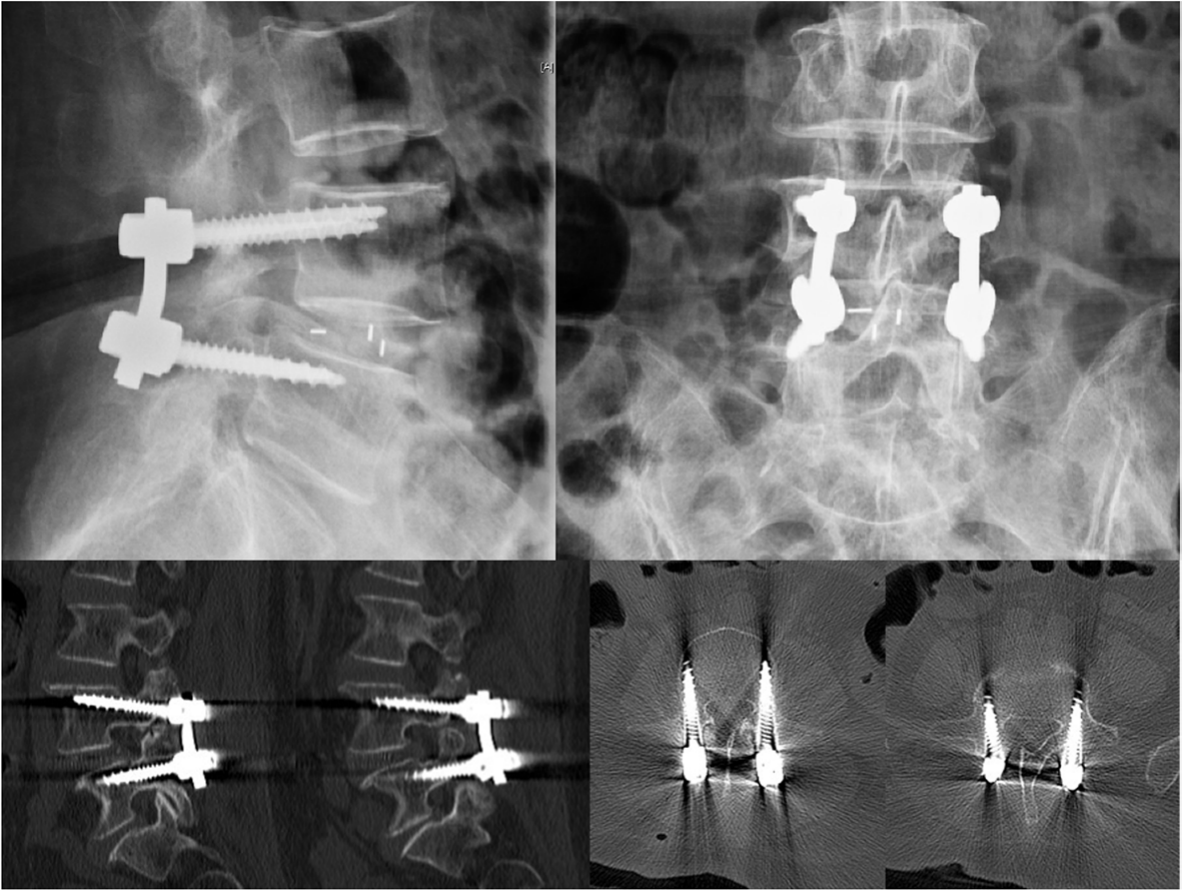
Postoperative reexamination of lumbar CT showed that all four screws reached grade 0 follow the score proposed by Ding et al. [12]

The findings of this study are presented in the following case examples (Fig. 4). A 72-year-old female previously diagnosed with lumbar spinal stenosis developed a 20-year history of lumbar spondylolisthesis that failed to respond to conservative treatment. The patient underwent CBT screw placement with robotic assistance at the L4 and L5 vertebral bodies, and the spinal canal was decompressed. The patient had no postoperative complications and was mobilized without a drain 4 days postoperatively. CT was performed on the fourth postoperative day (Fig. 5). The symptoms of lower-limb numbness and intermittent claudication were significantly relieved at the 1-year postoperative follow-up.

Several studies have shown that patients who undergo CBT have fewer postoperative complications and less pain than those who undergo the traditional pedicle screw technique [15–17]. Since the starting point of the screw is closer to the medial side, the length of the incision and the separation of the muscle are also reduced [18]. Compared with the traditional pedicle screw technique, biomechanical studies have also shown that a novel trajectory can improve the pullout load and insertion torque of CBT screws [19, 20]. However, its excellent biomechanical properties depend on the contact between the screw path and the multilayer cortical bone. Screw misplacement may affect the biomechanical properties of the screw and cause nerve injury [12]. Misplacement of screws has been associated with surgeon inexperience and incorrect screw entry point selection due to unclear anatomy. In fact, a cadaveric study by Crawford et al. [21] found that a small misdirection of as slight as 10° may cause a cortical wall breach.

In recent years, many related studies have been conducted on robot-assisted lumbar internal fixation placement. Jiang et al. [22] conducted a cohort study of 56 patients who underwent lumbar fusion of one-or two-level pedicle screws using Excelsius GPS (Globus Medical Inc., Audobon, Pennsylvania, USA). They found that patients who underwent robot-assisted fusion had less intraoperative blood loss and shorter hospital stay. Thus, they concluded that compared to fluoroscopy-assisted screw placement, robot-assisted screw placement can provide similar short-term outcomes. Le et al. [23] completed the placement of 86 CBT screws with robot assistance and compared them with screws placed with freehand. The robot used in this study was the same as the present study’s, which was TiRobot (TINAVI Medical Technologies Co., Ltd., Beijing, China). Their results indicated that robotic assistance was associated with a more satisfactory screw placement and reduced intraoperative radiation exposure. Another prospective cohort study by Zhang et al. [24] showed that compared with traditional open fluoroscopy-guided pedicle screw placement, robot-assisted percutaneous pedicle screw placement using the TiRobot system had fewer proximal facet joint violations, larger facet to screw distance, and higher intra-pedicle accuracy. In our study, compared to the fluoroscopy-assisted approach, screw placement accuracy with the robot-assisted technique was higher, violation of the superior facet joint was lower, and screw placement time and intraoperative radiation exposure time for the medical team were shorter.

The results of this study also showed that the trajectory of CBT screws was more accurate with robotic assistance than with fluoroscopy. To maximize cortical bone contact and improve purchase strength, the novel method of CBT relies on an accurate screw trajectory based on the four-point purchase between the dorsal cortex at the insertion site, the medial cortex of the posterior pedicle wall, the lateral cortex of the anterior pedicle wall, and the curvature of the vertebral body wall [19]. Therefore, the optimal biomechanical strength can only be obtained by ensuring the accuracy of the screw trajectory, indicating the importance of evaluating the screw trajectory accuracy. This study used a new screw trajectory evaluation system that is more suitable for evaluating the accuracy of CBT screw trajectories. This new method is consistent with the traditional evaluation method. Moreover, the modified system can help reduce the individual differences in the evaluation of cortical screw placement and can be used as a reference for the perfect insertion of cortical screws, increasing the evaluation reliability of rarer types of malpositions [12]. Le et al. [23] assessed CBT screw placement with TiRobot assistance using the Gertzbein scale [11], concluding that 95.3% of the screws reached an acceptable level, which was higher than that with fluoroscopy (86.9%) (*P* = 0.038). In the present study, the screw trajectory was evaluated using the novel method, and 93% of the screws in the robot-assisted group achieved a Grade 0 screw trajectory, as compared with the 83% of the screws in the fluoroscopy-assisted group. The difference between the two was statistically significant (*P* = 0.003).

The mean intraoperative fluoroscopy time was longer in the fluoroscopy-assisted group than in the robot-assisted group. Since intraoperative CT scanning is required when robot-assisted screw placement is used, intraoperative CT can improve the accuracy of screw trajectory, thereby shortening the time required for intraoperative fluoroscopy to obtain the ideal screw placement. This was consistent with the conclusions of previous studies [25]. However, this is only applicable to the medical team. Since the medical team can leave the operating room while performing intraoperative CT, their radiation exposure can only occur during intraoperative fluoroscopy. For patients, intraoperative robot-assisted screw placement necessitates an intraoperative CT scan with a much greater radiation dose than with intraoperative fluoroscopy alone. Thus, intraoperative radiation exposure for patients undergoing intraoperative robot-assisted screw placement is much greater than that for patients undergoing fluoroscopy-assisted surgery.

In addition, our study found that robot-assisted screw placement had a low rate of superior facet joint violation, that is, the distance between the facet and the screw, and the accuracy of the screw in the pedicle. Facet joint violation is one of the most common complications of posterior lumbar fusion and has attracted increasing attention in recent years. It has been reported to cause symptomatic adjacent segment disease and may affect the fusion rate of fusion surgery, resulting in low back pain [23]. Our study showed that robot-assisted screw placement can significantly (*P* < 0.05) reduce the invasion rate of superior facet joints and reduce the probability of superior facet joint degeneration.

Based on the learning curve associated with CBT described by Dayani et al. [26], a 6% medial perforation was reported in the early phase. Moreover, there was a trend towards statistical significance in the postoperative complications of 11 patients with 52 screws in the early experience group versus 11 patients in the late experience group. Dabbous et al. [27] found that the CBT technique can provide fixation comparable to that of the traditional PS technique; however, pedicle fracture and screw misplacement were significant risks during the learning curve of the new procedure. In a retrospective study of 80 consecutive patients by Kam et al. [28], robot-assisted pedicle screw placement had a very short (almost no) learning curve. The results of a retrospective study with a large sample size by Petrone et al. [29] also showed that accurate preoperative CT scan-based planning and the use of a patient-matched 3D template can significantly reduce the operation time while improving screw accuracy. They further stated that the use of these measures can reduce the learning curve of CBT techniques and make the advantage of less injury more evident. In our study, there was no statistically significant difference between the mean time required for the first 50% of screws and that required for the last 50% of screws by the same surgeon. In our center, the learning curve was almost zero when using Tirobot for CBT screw placement. By combining this with robot-assisted screw placement technology, the learning curve of CBT screw technology was noted to be shortened, and surgeons are expected to be able to master this new method more quickly.

Despite these findings, the study had some limitations. First, the lack of accurate radiation exposure dose data precludes an accurate assessment of the difference in patient radiation doses using robot-assisted or fluoroscopy-assisted screw placement. Second, long-term follow-up of patients with facet joint invasion was lacking. Third, many factors that can influence adjacent degeneration, such as the length of the fixed segment. Lastly, long-term follow-up is needed to confirm if robot-assisted screw placement does reduce the incidence of adjacent facet joint invasion, and therefore, adjacent degeneration.

## Conclusions

Robot-assisted screw placement can improve screw placement accuracy, shorten surgery time, effectively improve the safety and efficiency of surgery, and reduce radiation exposure to the medical team. Additionally, the difficulty and learning costs in robot-assisted screw placement are low.

## Data Availability

The data used and analyzed in this study are included in the article or are available from the corresponding and first authors upon reasonable request.

## Abbreviations

CBT: Cortical bone trajectory
RG: Robot-assisted
FA: Fluoroscopy-assisted
MRI: Magnetic resonance imaging
CT: Computed tomography

## References

[1] Santoni BG, Hynes RA, McGilvray KC, Rodriguez-Canessa G, Lyons AS, Hensons MAW (2009). Cortical bone trajectory for lumbar pedicle screws. Spine J.

[2] Su BW, Chaput CD (2015). Treatment of Spinal Conditions in Young Adults: Cortical Lumbar Screw Techniques. Oper Tech Orthop.

[3] Wochna JC, Marciano R, Catanesucu I, Katz J, Spalding MC, Narayan K (2018). Cortical Trajectory Pedicle Screws for the Fixation of Traumatic Thoracolumbar Fractures. Cureus.

[4] Cofano F, Marengo N, Ajello M, Penner F, Mammi M, Petrone S (2019). The Era of Cortical Bone Trajectory Screws in Spine Surgery: A Qualitative Review with Rating of Evidence. World Neurosurg.

[5] Zhang L, Tian N, Yang J, Ni W, Jin L (2020). Risk of pedicle and spinous process violation during cortical bone trajectory screw placement in the lumbar spine. BMC Muscoskelet Disord.

[6] Rexiti P, Aierken A, Sadeer A, Wang S, Abuduwali N, Deng Q (2020). Anatomy and Imaging Studies on Cortical Bone Screw Freehand Placement Applying Anatomical Targeting Technology. Orthop Surg.

[7] Levin JM, Alentado VJ, Healy AT, Steinmetz MP, Benzel EC, Mroz TE (2018). Superior Segment Facet Joint Violation During Instrumented Lumbar Fusion is Associated With Higher Reoperation Rates and Diminished Improvement in Quality of Life. Clin Spine Surg.

[8] Long J, Yu Y, Khan K, Li F, Zhu R, Zeng Z (2018). Superior Facet Joint Violations during Single Level Minimally Invasive Transforaminal Lumbar Interbody Fusion: A Preliminary Retrospective Clinical Study. Biomed Res Int.

[9] Fatima N, Massaad E, Hadzipasic M, Shankar GM, Shin JH (2021). Safety and Accuracy of Robot-Assisted Placement of Pedicle Screws Compared to Conventional Free-Hand Technique: A Systematic Review and Meta-Analysis. Spine J.

[10] Ringel F, Stüer C, Reinke A, Preuss A, Behr M, Auer F (2012). Accuracy of robot-assisted placement of lumbar and sacral pedicle screws: a prospective randomized comparison to conventional freehand screw implantation. Spine.

[11] Gertzbein SD, Robbins SE (1990). Accuracy of Pedicular Screw Placement In Vivo. Spine.

[12] Ding H, Han B, Hai Y, Liu Y, Guan L, Pan A (2021). The Feasibility of Assessing the Cortical Bone Trajectory Screw Placement Accuracy Using a Traditional Pedicle Screw Insertion Evaluation System. Clin Spine Surg.

[13] Yson SC, Sembrano JN, Sanders PC, Santos ERG, Ledonio CGT, Polly DW (2013). Comparison of Cranial Facet Joint Violation Rates Between Open and Percutaneous Pedicle Screw Placement Using Intraoperative 3-D CT (O-arm) Computer Navigation. Spine.

[14] Moshirfar A, Jenis LG, Spector LR, Burke PJ, Losina E, Katz JN (2006). Computed tomography evaluation of superior-segment facet-joint violation after pedicle instrumentation of the lumbar spine with a midline surgical approach. Spine.

[15] Keorochana G, Pairuchvej S, Trathitephun W, Arirachakaran A, Predeeprompan P, Kongtharvonskul J (2017). Comparative Outcomes of Cortical Screw Trajectory Fixation and Pedicle Screw Fixation in Lumbar Spinal Fusion: Systematic Review and Meta-analysis. World Neurosur.

[16] Snyder LA, Martinez-Del-Campo E, Neal MT, Zaidi HA, Awad A, Bina R (2016). Lumbar Spinal Fixation with Cortical Bone Trajectory Pedicle Screws in 79 Patients with Degenerative Disease: Perioperative Outcomes and Complications. World Neurosurg.

[17] Senoglu M, Karadag A, Kinali B, Bozkurt B, Middlebrooks EH, Grande AW (2017). Cortical Bone Trajectory Screw for Lumbar Fixation: A Quantitative Anatomic and Morphometric Evaluation. World Neurosurg.

[18] Mobbs RJ (2013). The “Medio-Latero-Superior Trajectory Technique”: an Alternative Cortical Trajectory for Pedicle Fixation. Orthop Surg.

[19] Matsukawa K, Yato Y, Kato T, Imabayashi H, Asazuma T, Nemoto K (2014). In vivo analysis of insertional torque during pedicle screwing using cortical bone trajectory technique. Spine.

[20] Phan K, Hogan J, Maharaj M, Mobbs RJ (2015). Cortical Bone Trajectory for Lumbar Pedicle Screw Placement: A Review of Published Reports. Orthop Surg.

[21] Crawford NR, Yüksel KZ, Doğan S, Villasana-Ramos O, Soto-Barraza JC, Baek S (2009). Trajectory analysis and pullout strength of self-centering lumbar pedicle screws. J Neurosurg Spine.

[22] Jiang B, Pennington Z, Azad T, Liu A, Ahmed AK, Zygourakis CC (2020). Robot-Assisted versus Freehand Instrumentation in Short-Segment Lumbar Fusion: Experience with Real-Time Image-Guided Spinal Robot. World Neurosurg.

[23] Le X, Tian W, Shi Z, Han X, Liu Y, Liu B (2018). Robot-Assisted Versus Fluoroscopy-Assisted Cortical Bone Trajectory Screw Instrumentation in Lumbar Spinal Surgery: A Matched-Cohort Comparison. World Neurosurg.

[24] Zhang Q, Xu Y, Tian W, Le X, Liu B, Liu Y (2019). Comparison of Superior-Level Facet Joint Violations Between Robot-Assisted Percutaneous Pedicle Screw Placement and Conventional Open Fluoroscopic-Guided Pedicle Screw Placement. Orthop Surg.

[25] Kumar KK, Parikh B, Jabarkheel R, Dirlikov B, Singh H (2021). Fluoroscopic versus CT-guided cortical bone trajectory pedicle screw fixation: Comparing trajectory related complications. J Clin Neurosci.

[26] Dayani F, Chen Y, Johnson E, Deb S, Wu Y, Pham L (2019). Minimally invasive lumbar pedicle screw fixation using cortical bone trajectory - Screw accuracy, complications, and learning curve in 100 screw placements. J Clin Neurosci.

[27] Dabbous B, Brown D, Tsitlakidis A, Arzoglou V (2016). Clinical outcomes during the learning curve of MIDline Lumbar Fusion (MIDLFA (R)) using the cortical bone trajectory. Acta Neurochir.

[28] Kam JKT, Gan C, Dimou S, Awad M, Kavar B, Nair G (2019). Learning Curve for Robot-Assisted Percutaneous Pedicle Screw Placement in Thoracolumbar Surgery. Asian Spine J.

[29] Petrone S, Marengo N, Ajello M, Lavorato A, Penner F, Cofano F (2020). Cortical bone trajectory technique’s outcomes and procedures for posterior lumbar fusion: A retrospective study. J Clin Neurosci.

